# Bond strength of dental nanocomposites repaired with a bulkfill composite

**DOI:** 10.4317/jced.53501

**Published:** 2017-03-01

**Authors:** Uzay Koç-Vural, Leyla Kerimova, İsmail H. Baltacioglu, Arlin Kiremitçi

**Affiliations:** 1 Postdoc Researcher, Hacettepe University, Department of Restorative Dentistry; 2 Research Assistant, Hacettepe University, Department of Restorative Dentistry; 3 Postdoc Researcher, Ankara University, Department of Restorative Dentistry; 4 Professor, Hacettepe University, Department of Restorative Dentistry

## Abstract

**Background:**

The aim of this study was to analyze the bond strength of aged resin based nanocomposites repaired with the same and bulk fill composites.

**Material and Methods:**

Seventy-two disc shaped resin composites consisted of three different nanocomposite resins (Filtek Ultimate/FU, Herculite XRV Ultra/HXRV, and Reflectys/R) were produced. After storing the samples for 8 weeks in distilled water, each material was combined with the same material or the bulk-fill composite resin system (Filtek Ultimate+Filtek Ultimate/Group-1; Filtek Ultimate+Tetric BF/Group-2; Herculite XRV+Herculite XRV/Group-3; Herculite XRV+Tetric BF/ Group-4; Reflectys+Reflectys/Group 5; Reflectys+Tetric BF/Group-6), for repair. Then specimens were subjected to shear bond strength testing(SBS), and the debonded surfaces were examined.

**Results:**

There was a significant difference among three materials(repaired with itself+bulk fill) for SBS testing values (*p*=0.001). FU and R were found to be similar, while HXRV was significantly different from them. A significant difference between group-1 and 2 (*p*=0.006) was detected, while there were no differences between group 3 and 4 (*p*= 0.142), and 5 and 6 (*p*=0.346). Among the six groups, repair SBS testing values with TBF were higher than repair with itself except for FU.

**Conclusions:**

The bulk-fill repaired materials showed higher bond strength except for FU, which showed the highest SBS value when repaired with itself. An increased incidence of adhesive fracture was observed at low strengths.

** Key words:**Resin-based composites, nanofillers, surface treatment, macro-shear, repair.

## Introduction

Dental resin based composites (RBCs), which are widely used in restoration of anterior and posterior teeth, still have limited lifetime. Dynamic changes in pH and temperature in the oral cavity due to diet, saliva and aging lead to degradation in the resin composite during clinical service (1,2). These changes can occur in various phenomena including micro-leakage, discoloration, wear, chipping, ditching or fracture and may lead to the replacement of the restoration (3,4). However, total replacement approach may weaken the tooth structure causing grinding sound tooth tissue or injuring the pulp tissue since in many cases remained restoration is clinically intact. Therefore, repairing the restorations may be another option for replacement (5-7).

The success of the repair, performed by using a resin composite, depends on many factors including surface characteristics (7), wettability (8) roughness (9), and the surface conditioning methods performed (7,10,11). To improve the adhesion between aged and non aged composite resins, different surface conditioning methods were developed and surface conditioning has an important impact on repair bond strength because the adhesive strength of composite to composite restorations decreases by 25% to 80% compared to their original strength (12,13).

Different surface conditioning methods, including increased surface roughness (12,14) silane treatment (10), and the application of bonding agent (6,10) were investigated in the literature. It has been reported that, increased surface roughness is required to promote micromechanical interlocking between the composite surfaces (5). Surface grinding using an abrasive tool prior to repair is considered to be the simplest method for preparing the surface of the composite in daily clinical practice.

Many studies were conducted to reach a consensus on the aging technique representing the closest to the relevant clinical scenario for composite-to-composite bonding (3,6,15) including thermocycling (16), water storage (17,18), citric acid immersion (11) and boiling (18). When a resin composite is subjected to the water storage, some damaging effects will occur on the resin composite surface including both hydrolysis and release of filler particles besides water absorption into the resin matrix (19,20).

The objective of the present study was to compare the repair bond strengths of nano hybrid and bulk-fill resin composites after water storage. The tested null hypothesis was that the repair bond strength would not decrease in any types of composite resins.

## Material and Methods

The compositions and manufacturer details of used nanocomposites are listed in  Table 1.

**Table 1:**
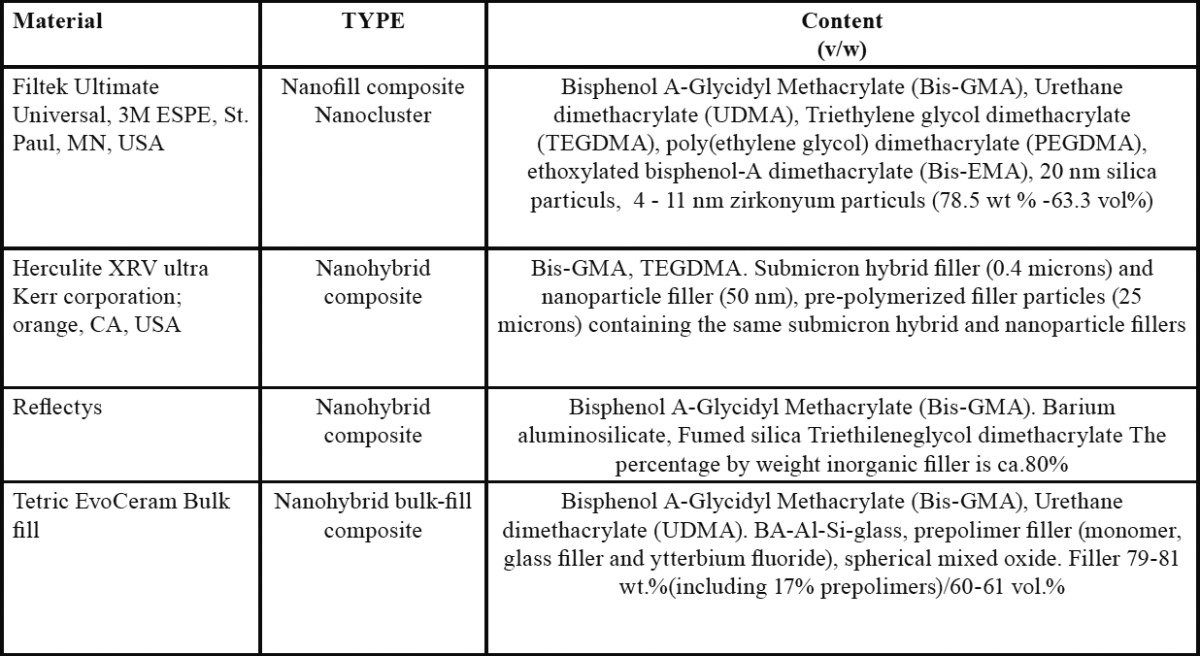
Materials, manufacturer, and chemical composition.

**Figure 1:**
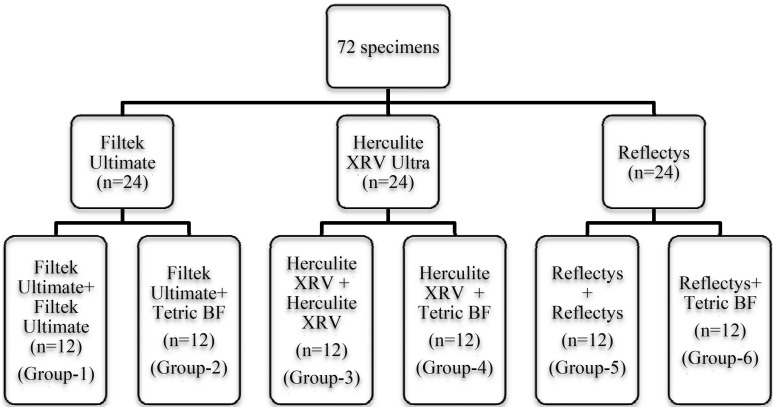
Flow diagram of the experimental design.

Seventy-two disc shaped resin composites consisted of three different nano-composite resins (Filtek Ultimate Ultimate (FU), Herculite XRV Ultra (HXRV), and Reflectys (R) ) were produced to evaluate the repair bond strength by filling the teflon cylinders in 3.5 mm thickness and 6 mm diameter. The composites were carefully condensed with a clean filling instrument in order to avoid any contamination. The composite discs were cured in 2 mm layers with layering technique using a LED light device (Mega-Physik, Cromalux 1200, 1400mW/cm2, Rastatt Germany). A Mylar strip and glass slide were used at both ends of the Teflon mold to achieve flat-ended specimens. Each of the three materials used in the present study was assigned as a filling (substrate) material and each substrate had 24 specimens. Immediately after the polymerization, the specimens were stored in distilled water at 37ºC for 8 weeks. The water was changed every week to prevent growth of bacteria. Before the subsequent filling repair, one of the surfaces of each aged 72 specimens (n=24) belonged to three experiment groups was roughened with a 320-grit silicon carbide sandpaper under running water for 5 seconds to obtain a standardized roughened surface. All experimental specimens were covered with 37% phosphoric acid gel (Condac 37, FGM, Setubal, Portugal) for 15 s, rinsed with water for another 15 s, and dried with compressed air. The intermediate bonding agent (Prime & Bond NT, Dentsply De Trey; Konstanz, Germany) was applied and polymerized according to the manufacturers’ recommendations for placement of composite restorations. Each experimental group was divided into subgroups so that each material was combined with itself or bulk-fill composite resin system for repair. The repair composite to be tested was applied by means of a Teflon jig (Ultradent Products Inc., South Jordan, UT) in two separate increments (2.38 mm in diameter, 2.5 mm in height) and polymerized for 20 s. Thus, 6 combinations (n=12) resulting in 72 specimens were made available for testing (Fig. 1).

The specimens were again stored in distilled water at 37ºC for 24 h. Then, the specimens were transferred to an Instron testing machine (Lloyd, Model LRX, England) and subjected to shear bond strength (SBS) test. The specimens were positioned in the device so that the shearing stamp would load the composite cylinder at a 90° angle with a crosshead speed of 1mm/s and a cell load capacity of 1 kN until failure. After the break, the surfaces were examined under a stereomicroscope (American Optical, Buffalo, NY, USA) at 40X magnification to determine the exact type of fracture and classify whether it is adhesive, cohesive and mixed. Fracture in the composite was classified as cohesive failure. If the residues of either the adhesive and/or the composite had been detectable, the specimen would have been assigned to the group of mixed fractures/failure. Adhesive fracture between the adhesive agent and the composite was classified as adhesive failure.

Statistical analysis was performed using the software SPSS 21.0 for MAC and ANOVA. Mc-Nemar and the Bonferroni post hoc tests were performed. A *p* value <0.05 was considered as statistically significant.

## Results

There were no pre-testing failures among the specimens.

There was a significant difference between three materials (repaired with itself + bulkfill) for SBS testing values (*p*=0.001). FU and R were found to be similar, while HXRV was significantly different. Besides, HXRV showed the lowest SBS values among the groups.

There was a significant difference between group 1 and 2 (*p*=0.006), while there were no differences between group 3 and 4 (*p*= 0.142), and 5 and 6 (*p*=0.346) ( Table 2). FU repaired with the same kind, presented the highest SBS values.

**Table 2:**
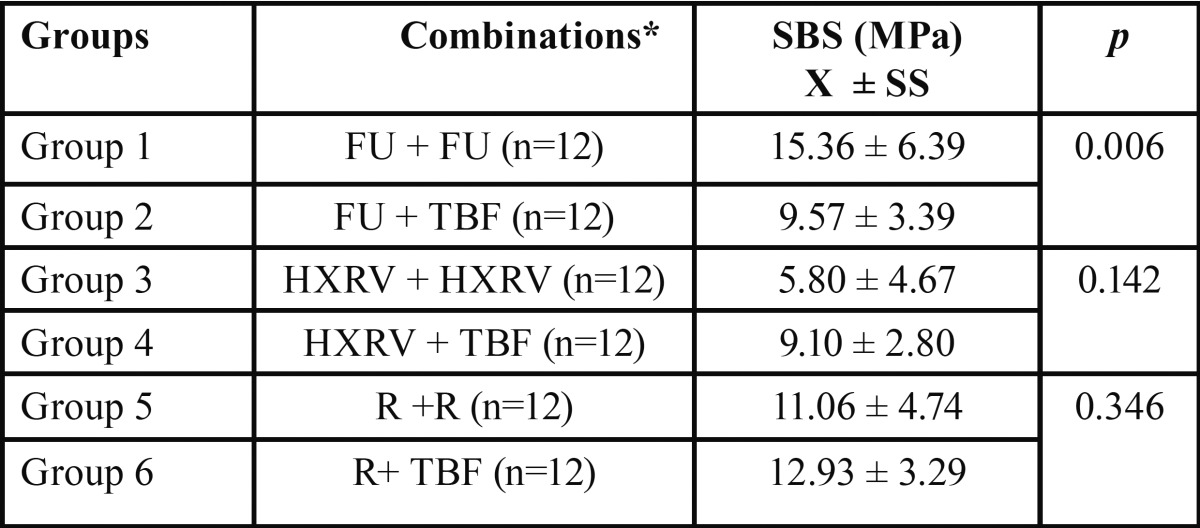
Mean SBS test values of the groups.

Among the six groups, repair SBS testing values with TBF were higher than repair testing values with itself except for FU.

The results of the shear bond strength tests of repaired composites are summarized in  Table 2.

The highest bond strength was observed in Group 1 and 6, while the lowest was observed in groups 2, 3, and 4. Results of fracture mode analyses were shown in  Table 3.

**Table 3:**
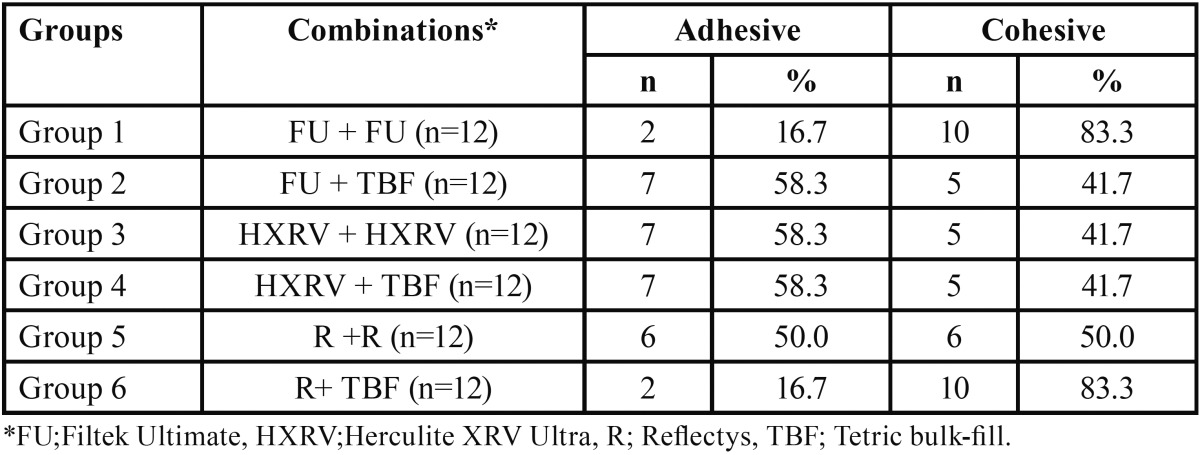
Results of fracture mode analyses.

## Discussion

Resin based composites (RBCs) with different formulations are popular materials in dentistry. However, they have some complications such as polymerization shrinkage, lack of adaptation to cavity walls, microleakage or cracks (21-23). To overcome these complications the size of filler particles was decreased and incremental layering technique was proposed. Nanocomposites, including nanofiller or nanohybrid compositions, contain filler particles in the range of 0.1-100 nm to obtain an improved wear resistance, high fracture toughness, optimum smoothness and aesthetics smoothness (24). However, the incremental layering technique has some disadvantages including the contamination or bond failure risk between the composite layers, difficulties in adaptation of resin composites into the conservatively prepared cavities as well as increased application time (25). The bulk-fill materials, which claim to minimize the polymerization shrinkage stress and time consumption with a depth cure in an excess of 4 mm, were introduced to minimize the aforementioned disadvantages. According to the manufacturers, the reduced filler particle size shows slow polymerization that results with less polymerization shrinkage stress. Currently, bulk-fill composite materials become increasingly popular among dental practitioners (26). However, in the literature, only a little attention was paid to the repair of aged composite with bulk fill composite. To best of our knowledge, there was no study recently published aiming to discover SBS of bulk-fill composites to different nanocomposites. In a clinical situation when a composite is to be repaired, the operator is unable to identify the brand or type of the old composite. Because of this reason, two clinical scenarios were simulated; one was the situation when the repair material was the composite’s itself and the second was the situation when the repair material was the bulk-fill resin composite.

When a restoration requires repair, it means that the composite resin has reached to the highest level of water saturation, which causes softening of the matrix, micro-crack formation, resin degradation and debonding of the filler-matrix interfaces. As a result free radical activity has ended and oxygen inhibited layer was disappeared (27). However, aged substrate needs to be activated either chemically or physicochemically.

In the literature, there are no common instructions for the aging regimens simulating the oral conditions. However, water storage is the most commonly used aging approach among different methods including thermal cycling and boiling. In this study, the specimens were stored in water for 8 weeks to obtain an aged substrate surface (11,16) which contains diminished radical activity of monomer functional groups.

According to the results of this study, in aged conditions, both of the nano-composites behaved similarly, while HXRV was different from them. HXRV showed significantly decreased repair strength. This could be explained by the fact that nanofilled FU contains bis- GMA, UDMA, TEGDMA and PEGDMA and nanohybrid R contains only Bis-GMA, while nanohybrid HXRV contains a resin matrix composed of bis- GMA and TEGDMA. A rigid cross-linked network produced by bis-GMA (1.43 GPa) absorbs less water than TEGDMA does, but more water than UDMA does. TEGDMA is a hydrophilic monomer, that absorbs great amount of water (20,28).

On the other hand, between the six subgroups, FU showed significantly increased bond strength when it was combined with itself. However, in all groups, repair bond strength with TBF was similar or better than the repair bond strength of nanohybrid resin composites. This could be explained by the fact that FU is a nanofilled composite that contains 20 nm silica particles, 4 - 11 nm zirconium particles, while others are nanohybride. The nanofilled resin composites include the nanosized particles throughout the resin matrix (29), while the nanohybrid resin composites combine the both nanomeric and conventional fillers (30,31) and this characteristic is similar to microhybrid composites. From this point of view, observing increased bond strength when the repair material is the same as the material of the composite rather than when the repair material is a different type of composite is reasonable. However, repairing with the same material is a proposed approach in the literature.

Also, in the literature it was shown that, nanofilled RBCs still exhibit problems in the long term usages, such as secondary caries and fractures (32-34). In this study, FU showed better results among the tested materials and correlates with the current literature. Besides, in correlation with our study, Ilıe N *et al.* (35) investigated the shear bond strength of bulk-fill resin composites of permanent and deciduous teeth and reported that bulk-fill materials including Tetric Evo Ceram, performed similar or better than the shear bond strength of nanohybrid resin composites.

The tested null hypothesis stating that repair materials would not decrease the repair bond strength in any type of composite can only be accepted partially. Remarkable results were found in this study for HXRV. The highest number of adhesive fracture occurred in this group and the repair strength showed significantly less SBS values than those of FU and R with both of the repair methods. To the best of our knowledge, there was no so many studies investigated the repair bond strength of HXRV. Moezzyzadeh (36) investigated compressive strength of hybrid and nanocomposites and reported that hybrid composites were superior than nanocomposites including HXRV. Papacchını *et al.* (37) investigated the contribution of silane to the HXRV resin composite repair strength over time and reported no absolute advantage in using silane for repairing HXRV composite however, their results can not comparable with the present study because of used technique (38).

## Conclusions

The repair bond strength is affected mostly by the substrates. These circumstances make the success or failure of the repaired restoration to be difficult to foresee, because the operator rarely knows the used substrate. However, bulk-fill repaired substrates used in all combinations resulted with a satisfying bond strength proving that these materials are clinically applicable, and the bond strengths correlated with fracture mode patterns. The incidence of adhesive fracture was increased at low strengths.
